# Development of a Mobile App to Increase the Uptake of HIV Pre-exposure Prophylaxis Among Latino Sexual Minority Men: Qualitative Needs Assessment

**DOI:** 10.2196/43844

**Published:** 2023-02-08

**Authors:** Valeria D Cantos, Kimberly Hagen, Ana Paula Duarte, Carolina Escobar, Isabella Batina, Humberto Orozco, Josue Rodriguez, Andres Camacho-Gonzalez, Aaron J Siegler

**Affiliations:** 1 Division of Infectious Diseases Emory University School of Medicine Atlanta, GA United States; 2 Department of Behavioral, Social and Health Education Sciences Rollins School of Public Health Emory University Atlanta, GA United States; 3 Centers for Disease Control and Prevention Atlanta, GA United States; 4 NorthShore University HealthSystem Evanston, GA United States; 5 Emory University School of Medicine Atlanta, GA United States; 6 Department of Pediatrics Emory University School of Medicine Atlanta, GA United States

**Keywords:** pre-exposure prophylaxis, PrEP, Latino, sexual minority men, SMM, mobile health, mHealth, HIV prevention, HIV, technology, minority, men, development, biomedical intervention, mobile technology, mobile app, community, app, barrier, mobile phone

## Abstract

**Background:**

HIV disproportionally impacts Latino sexual minority men (SMM). Uptake of pre-exposure prophylaxis (PrEP), an effective biomedical intervention to prevent HIV, is low in this group compared with White SMM. Mobile health technology represents an innovative strategy to increase PrEP uptake among Latino SMM.

**Objective:**

We aimed to describe the qualitative process leading to the development of SaludFindr, a comprehensive HIV prevention mobile app aiming to increase PrEP uptake, HIV testing, and condom use by Latino SMM.

**Methods:**

We conducted 13 in-depth interviews with Latino SMM living in the Atlanta area to explore their main barriers and facilitators to PrEP uptake and to analyze their opinions of potential SaludFindr app functionalities. To explore potential app functions, we used HealthMindr, an existing HIV prevention app, as a template and added new proposed features intended to address the specific community needs.

**Results:**

We identified general PrEP uptake barriers that, although common among non-Latino groups, had added complexities such as the influence of religion and family on stigma. Low perceived PrEP eligibility, intersectional stigma, lack of insurance, cost concerns, and misconceptions about PrEP side effects were described as general barriers. We also identified Latino-specific barriers that predominantly hinder access to existing services, including a scarcity of PrEP clinics that are prepared to provide culturally concordant services, limited availability of Spanish language information related to PrEP access, distrust of peers as credible sources of information, perceived ineligibility for low-cost services owing to undocumented status, fear of immigration authorities, and competing work obligations that prevent PrEP clinic attendance. Health care providers represented a trusted source of information, and 3 provider characteristics were identified as PrEP facilitators: familiarity with prescribing PrEP; being Latino; and being part of lesbian, gay, bisexual, transgender, queer, intersex, and asexual (LGBTQIA+) group or ally. The proposed app was very well accepted, with a particularly high interest in features that facilitate PrEP access, including a tailored list of clinics that meet the community needs and a private platform to seek PrEP information. Spanish language availability and free or low-cost PrEP care represented the 2 main clinic criteria that would facilitate PrEP uptake. Latino representation in clinic staff and providers; clinic perception as a safe space for undocumented patients; and LGBTQIA+ representation was listed as additional criteria. Only 8 of 47 clinics listed on the Centers for Diseases Control and Prevention PrEP locator website for the Atlanta area fulfilled at least 2 main criteria.

**Conclusions:**

This study provides further evidence of the substantial PrEP uptake barriers that Latino SMM face; exposes the urgent need to increase the number of accessible PrEP-providing clinics for Latino SMM; and proposes an innovative, community-driven, and mobile technology–based tool as a future intervention to overcome some of these barriers.

## Introduction

### Background

Latino sexual minority men (SMM), including gay, bisexual, and other men who have sex with men, are disproportionally affected by HIV in the United States [1,2]. This group, particularly those living in the South [3,4], is especially vulnerable to HIV owing to high group prevalence stemming from social and structural factors, including language barriers, poverty, discrimination, social isolation, low rates of health insurance, and ineligibility for federally funded insurance [3,5-9]. Furthermore, heightened societal anti-immigrant sentiment and intensified law enforcement practices may create a “chilling effect,” pushing Latino SMM further away from facilities offering pre-exposure prophylaxis (PrEP) and other HIV prevention services [10]. PrEP uptake among Latino SMM is substantially lower than that among White SMM [11], with only 14% of PrEP-eligible Latina/o/x (henceforward “Latino”) individuals being prescribed PrEP in 2020 compared with 61% of White SMM [12]. Given this disparity, it is critical to develop methods to reach Latino SMM. Most Latino SMM own a smartphone and use it as their primary internet access [13,14], making mobile health (mHealth) technology a promising tool for overcoming PrEP uptake barriers in this population. Specific advantages of mHealth include scalability, the ability to accommodate multiple language needs, and the nonstigmatizing nature of private web-based spaces [15-20].

A growing number of evidence-based HIV prevention mobile apps for SMM have been developed [16,17,21,22]. HealthMindr [18], a comprehensive HIV prevention app, features self-assessments that direct users to appropriate services, including an HIV testing or PrEP service locator; mail delivery of condoms, lubricant, and home test kits for detecting HIV infection or sexually transmitted infections (STIs); and customizable HIV testing and PrEP care maintenance plans [18]. During a 4-month pilot test, HealthMindr reached an “excellent” system usability score of 73.4 [23]. Service use was high despite the lack of incentives for use; 65% of the participants ordered condoms, 54% ordered home HIV testing kits, 72% used the app’s HIV testing plan, and 9% (of PrEP-eligible individuals) started taking PrEP, with 75% of them reporting that the app influenced their decision [18,24,25]. Other eHealth methodologies, including SMS text messaging and website-based platforms, have also been proposed to address HIV and STI prevention. PrEPmate, an interactive SMS text messaging intervention, was successful in increasing PrEP retention and adherence among primarily Black and Latino youth, when compared with the standard of care during the 36 weeks of study participation [26]. Mustanski et al [27] developed a 7-module, web-based, multimedia intervention (Keep it Up!), which resulted in lower STI incidence compared with a web-based HIV knowledge condition [27].

### Objectives

To our knowledge, no comprehensive HIV prevention app has been tailored to Latino SMM and no evidence-based and publicly available HIV prevention apps are available in Spanish language versions. Following a modified version of the Assessment, Decision, Adaptation, Production, Topical Experts–Integration, Training, Testing (ADAPT-ITT) framework, a theoretical model of 8 sequential steps used to adapt HIV prevention evidence-based interventions [28], we first decided to use HealthMindr as a template app for the creation of SaludFindr, an HIV prevention app for Latino SMM. We then conducted a needs assessment through in-depth interviews (IDIs) with Latino SMM. These interviews discussed PrEP uptake facilitators and barriers, solicited thoughts about the key functions of an app specifically developed for Latino SMM, asked their opinion of the existing HealthMindr functionalities, and explored their ideas for new potential SaludFindr features. According to Muessig et al [16], addressing specific community’s needs and preferences must precede app development to allow for the needs to be incorporated into the app development process. This paper presents the results of 13 IDIs conducted as part of the ADAPT-ITT framework with Latino SMM living in the Atlanta Metropolitan Area.

## Methods

### Study Population and Recruitment

We conducted 13 IDIs between December 2019 and March 2020. We recruited Latino SMM aged ≥18 years through web-based social media advertisements and venue-based active recruitment activities. Eligibility criteria were as follows: (1) aged ≥18 years, (2) Latino by self-report, (3) gay or bisexual by self-report, (4) currently living in the Atlanta Metropolitan Area, (5) never received a positive HIV test, and (6) owns a smartphone.

Study recruitment occurred electronically and in person. English and Spanish advertisements were posted on Facebook and Instagram. Men who clicked on the advertisement were directed to a link containing information about the study and a web-based informed consent form for screening. Consenting participants completed an electronic eligibility and sociodemographic survey. Eligible men provided contact information and their preferred mode of contact. The research staff contacted the participants to schedule their IDI. Through existing local community partnerships, the research staff attended events at popular clubs among the Latino SMM groups in the area, where we distributed physical advertisements and engaged in conversations with attendees about the study. Interested individuals completed the electronic informed consent form for screening and the eligibility survey on site and were later contacted by the research staff to schedule the IDI.

All study materials (consents, surveys, and IDI guide) were available in English and Spanish, and we accommodated language preferences in conducting the IDI. Study reporting followed the COREQ (Consolidated Criteria for Reporting Qualitative Research) guidance [29]. Before starting the IDI, the research staff conducted a verbal consent process with participants, informing them about the purpose of the study, the study procedures, and the potential risks and benefits of their participation. After IDI completion, the participants received a US $25 gift card. The study data were deidentified before the analysis.

### Ethics Approval

This study was approved by the Emory University Institutional Review Board (IRB: IRB00097743).

### Study Procedures

#### Overview

For this study, we used the ADAPT-ITT framework (Textbox 1) [28]. We first examined the available literature to decide which evidence-based intervention should be used as a template app to develop SaludFindr (ADAPT-ITT phase “Decision”). We opted to use the HealthMindr intervention because it had strong preliminary evidence of impact and it matched our targeted primary outcomes (PrEP uptake, HIV testing, and increased condom use) and general target population (SMM).

**Overview of study procedures.**

**Assessment, Decision, Adaptation, Production, Topical Experts–Integration, Training, Testing (ADAPT-ITT) phase: study procedure**

1. Decision: Examination of the literature to decide to use HealthMindr as template app
2. Assessment: In-depth interviews with Latino sexual minority men (SMM) to inform the adoption or adaptation of HealthMindr for SaludFindr

#### Needs Assessment (ADAPT-ITT Phase: “Assessment”)

To determine whether HealthMindr would be either adopted as it was originally created or adapted (and if so, how it should be adapted) for the Latino SMM population, we conducted a needs assessment with 13 Latino SMM through IDIs. We offered the option of conducting the IDIs in Spanish or English. Approximately two-thirds (8/13, 61%) of the participants preferred to perform the IDIs in Spanish. We also offered the option of in-person or web-based interviews. Almost all participants (11/13, 85%) preferred the web-based option. For conducting the web-based interviews, we used Zoom (Zoom Video Communications), a secure, web-based audio- and videoconferencing platform. The IDIs lasted for approximately 1 hour. Study staff members (VDC, APD, and CE), all trained in qualitative research, conducted the IDI.

During the IDI, we assessed participants’ thoughts on major PrEP uptake facilitators and barriers for Latino SMM living in the Atlanta area. We then gave a short presentation of the app using pictures of the original HealthMindr features along with a verbal description of its functionalities. Participants expressed their opinions of each feature’s usefulness, preferred language and tone, and suggestions to optimize usability. We also explored participants’ opinions on potential additional app features that are not currently available in HealthMindr. Latino LinQ, our partnering community-based organization (CBO), proposed creating a tailored list of *Latino- and gay-friendly* sexual health clinics to facilitate PrEP access. For this purpose, we asked participants to describe what characteristics they would look for in a clinic, focusing on what would make them feel at ease and most likely to attend. On the basis of their responses, we established criteria to define a clinic as *Latino and gay friendly* and created a checklist of these criteria. Other new potential app functionalities included tailored messages delivered through app push notifications to motivate PrEP uptake using a sex-positive approach and a platform to discuss HIV- and PrEP-related topics. We also sought inductive participant feedback, probing whether there were other HIV prevention app features or functions that would be useful for the community.

Finally, we assessed the potential safety and privacy concerns related to the app, overall app acceptability, the relative importance of developing an app specifically for Latino SMM, and how the app would address the main facilitators and barriers to PrEP uptake in the Latino community.

### Data Analysis

The IDIs were digitally recorded in audio files, transcribed verbatim, and deidentified. We used a modified version of grounded theory to code and analyze the IDIs. Once transcribed, 2 study team members (VC and AD) independently reviewed 20% of the total number of transcripts to generate preliminary codes and code definitions using inductive and deductive methods. This process continued until saturation of codes was reached (ie, when no team member identifies new codes). Once the codebook was developed, 20% of the transcripts were independently coded by 2 team members for intercoder agreement (AD and KH). After independent coding, coders met to discuss their analysis and conflicts were resolved by consensus between the coders. After consensus was reached, each coder coded the remaining transcripts. We then created memos to describe and verify the relationships among the most salient themes. Major themes and relationships across all the participants were consolidated into a narrative. All analyses were conducted using MAXQDA (version 12; VERBI Software GmbH).

## Results

### Participant’s Characteristics

The median age of the participants was 30 years, and all identified as cisgender men. Most participants were foreign-born (9/13, 69%; from Mexico and Central and South American countries), and more than two-thirds (6/9, 67%) of them arrived in the United States at the age of ≥13 years. Of the 13 participants, 6 (46%) were currently on PrEP (Table 1).

**Table 1:** Baseline participant characteristics (N=13).

Characteristics			Values
Age (years), median (IQR)			30 (22-37)
Sex at birth (male), n (%)			13 (100)
**Educational assessment, n (%)**			
	High school		2 (15)
	College or more		7 (54)
	Did not respond		4 (31)
**Ever tested for HIV, n (%)**			
	Yes		10 (77)
	No		1 (8)
	Did not respond		2 (15)
**HIV status, n (%)**			
	Negative		10 (77)
	I do not know		1 (8)
	Did not respond		2 (15)
**Documentation status, n (%)**			
	DACA^a^		2 (15)
	Permanent resident		1 (8)
	US citizen		2 (15)
	Visa		3 (23)
	Undocumented		1 (8)
	Did not respond		4 (31)
**Country of origin, n (%)**			
	US born		1 (8)
	**Foreign-born**		9 (69)
		Arrived to the United States at age 12 years or younger	3 (33)
		Arrived to the United States at age 13 years or older	6 (67)
	Did not respond		3 (23)
**Country of origin, n (%)**			
	Brazil		1 (8)
	Colombia		2 (15)
	Guatemala		1 (8)
	Mexico		2 (15)
	Peru		1 (8)
	Venezuela		1 (7)
	Other or did not respond		5 (39)
**Interview language, n (%)**			
	Spanish		8 (61)
	English		5 (39)
**PrEP^b^ uptake, n (%)**			
	Never on PrEP		7 (54)
	Currently on PrEP		6 (46)

^a^DACA: Deferred Action for Childhood Arrival.

^b^PrEP: pre-exposure prophylaxis.

### PrEP Uptake Barriers and Facilitators

Participants reported multiple general PrEP uptake barriers including (1) low perceived PrEP eligibility; (2) intersectional stigma (HIV, gay, and PrEP related); (3) lack of insurance or perceived cost; and (4) PrEP misconceptions, which are mostly about side effects. For some of these factors, there were added complexities that were specific to Latino groups. For example, some participants noted how the influence of religion and family, both very important in Latino culture, makes gay-related stigma even more challenging to overcome:

So that’s probably the biggest challenge with these kind of things because, as gays, we try to keep some things under the radar. But then being Latin, it creates like more pressures, because of your family and the background that we have in the countries.

IDI participant, English, aged 37 years

Several Latino-specific barriers were identified. All participants agreed that there is a scarcity of reliable Spanish language information about PrEP and how to access it. Most participants who were not on PrEP (7/13, 54%) had received PrEP-related information from friends in the context of rumors or *chisme* (gossip) or through social media. Both sources were deemed unreliable by participants and hence not conducive to facilitating PrEP care seeking or access. Some IDI participants (6/13, 46%) discussed the scarcity of open conversations around sexual health (HIV status, HIV testing, condoms, or PrEP) in social settings among friends, mostly because of the abovementioned multifactorial stigma. As a result, there is very limited sharing of potentially useful information about PrEP—those who are on PrEP tend to hide it from the rest and those who are not on PrEP but would like to know more about it do not seek guidance from their peers:

I think we need more programs that focus on how we can talk among ourselves...it’s not very Latino to air out everything that’s going on in your personal life.

IDI participant, Spanish, aged 30 years

Another group of PrEP uptake barriers was related to the challenges of accessing existing sexual health services, exacerbated by participants’ immigration status. These include (1) language barriers, which make seeking PrEP information and interacting with clinic staff difficult; (2) assumed ineligibility for free or low-cost PrEP solely because of their undocumented immigration status; (3) fear of immigration law enforcement and deportation; and (4) competing work obligations. Many participants reported working in informal job positions (construction, restaurants, and mechanics) in which payment is contingent on work attendance, and it is difficult to obtain permission to take time off to come to a clinic:

So we have the issue of the legal status of the Latin community. So because of that, a lot of the guys that I know, sometimes because they don’t have a legal status inside the country, so they think they can’t have—they’re not allowed to have access to some prevention programs.

IDI participant, English, aged 37 years

Participants reported a few PrEP facilitators. First, most participants agreed that physicians’ opinions on PrEP benefits and potential risks represented a credible source of information on which decisions about starting PrEP could be made. The participants highlighted three provider characteristics that facilitate PrEP uptake: (1) familiarity with prescribing PrEP; (2) being Latino (or at least fluent in Spanish); and (3) being part of lesbian, gay, bisexual, transgender, queer, intersex, and asexual (LGBTQIA+) groups or an ally. Participants explained that these 3 features would also make conversations surrounding PrEP easier as they would feel more comfortable with someone who understands who they are and the social complexities they endure from being gay or bisexual Latino men (eg, discrimination, social and family rejection, and homophobia). Related to the challenges of accessing PrEP care, some participants suggested flexible clinic hours and the use of technology in the form of emails and SMS text messages to communicate with clinic staff, as these would facilitate access for those who are not able to attend in-person clinic visits during working hours.

One participant summarized the complexity of multiple PrEP uptake barriers that Latino SMM can face, such as stigma, health insurance, PrEP misinformation, and challenges of legal status:

Because most people work and have no time to go, because they don’t have health insurance, because they don’t have money to pay, well, also because of embarrassment because there’s also a lot of people who have-there’s a lot of stuff they put on Facebook, they put ads that PrEP is bad, that, for example, it damages the bones, or damages the liver, or damages the kidneys, because using PrEP has its consequences. Also, many don’t use it out of fear. But I think that basically, it’s because they don’t have health insurance, and also, Latinos who don’t have papers, who are undocumented, well they don’t have-it’s hard to get it. Well, I think those are the main reasons. But also because they are embarrassed.

IDI participant, Spanish, aged 45 years

Table 2 provides a summarized list of PrEP uptake facilitators, shared PrEP uptake barriers with non-Latino SMM, and specific Latino SMM–related barriers.

**Table 2:** Thematic analysis of pre-exposure prophylaxis (PrEP) uptake facilitators and barriers.

Theme				Values, n^a^		Participants’ quotes
**Theme 1: general PrEP uptake barriers (N=13)**						
	Low self-perceived PrEP eligibility		2		“I’m not that interested in that pill because I don’t feel like I need it.” [IDI participant, Spanish, aged 22 years]	
	Intersectional stigma: gay, HIV, and PrEP related		5		“I hate to generalize, but I just feel like it’s worse in my culture, the Latino culture, than it is other cultures. They don’t understand the prevention aspect of it. So, they automatically assume you are HIV positive and, therefore, you are diseased, you’re dirty, you’re going to give it to them, you’re sexually fluid because you must be then gay, and that automatically, to them, means that you have no morals.” [IDI participant, English, aged 44 years]	
	Lack of insurance or perceived cost		9		“Because we’re in the US, everything is expensive, especially for those who don’t have papers or insurance. The problem is to afford the medication, it’s very expensive.” [IDI participant, Spanish, aged 45 years]	
	PrEP misconceptions, mostly related to side effects		8		“I’ve heard there are side effects, related to warts and I think a type of cancer.” [IDI participant, Spanish, aged 22 years]	
**Theme 2: PrEP uptake barriers that predominantly impact Latino SMM^b^ (N=13)**						
	Scarcity of trustworthy PrEP information sources, especially in Spanish		7		“I definitely see more advertisement about stuff like this in English. We are an English-speaking country, so I understand that. But even in like places that would be Spanish-speaking or Portuguese-speaking, I don’t see the advertisements for it. I wish there were more.” [IDI participant, English, aged 44 years]	
	Avoidance of PrEP-related discussions among peers		6		“I think we need more programs that focus on how we can talk among ourselves...it’s not very Latino to air out everything that’s going on in your personal life.” [IDI participant, Spanish, aged 30 years]	
	**Challenges accessing existing sexual health services subcode**					
		Language barriers	3		“Payment, misinformation, and English barrier. You know? I think those would be the biggest issues.” [IDI participant, English, aged 36 years]	
		Assumed ineligibility for low-cost services because of undocumented status	9		“We have the issue of the legal status of the Latin community. So because of that, a lot of the guys that I know, sometimes because they don’t have a legal status inside the country, so they think they can’t have—they’re not allowed to have access to some prevention programs.” [IDI participant, English, aged 26 years]	
		Fear of immigration law enforcement and deportation	6		“You don’t want to try to help your health and then get locked up in jail or deported and separated from your family and friends. So, you’d rather just not even talk about it or do it or go to it.” [IDI participant, English, aged 44 years]	
		Competing work obligations	6		“We work a lot when we’re here,...when the clinics are open, they can’t go, because they can’t just ask for a day off. You make $400.00 a week, and you need to skip a day to go see a doctor, and your boss is going to say like this. So why are you going to see a doctor? Oh, I want to take PrEP. It’s not because you’re sick or something. They’re going to say no.” [IDI participant, English, aged 36 years]	
**Theme 3: PrEP uptake facilitators (n=7)**						
	Knowing a health care provider who has familiarity providing PrEP		2		“I spent like one or two years with one or two doctors who didn’t know about PrEP, until I finally found a doctor who knew and he was the one who helped me.” [IDI participant, Spanish, aged 45 years]	
	Having a health care provider is part of LGBTQIA+^c^ groups or ally		1		“She just immediately told me, ‘I just want you to know I’m an ally and I have many gay friends and I grew up with gay people. So, I want you to know you’re safe with me. You’re comfortable. I think it’s excellent that you want to learn more about the medication and get on it and I’m going to help you do that.’ So, she did.” [IDI participant, English, aged 44 years]	
	Having a health care provider who’s a Latino		1		“I think most people would agree that they would feel more comfortable if the person actually was Latino as well.” [IDI participant, English, aged 44 years]	
	Trust in health care providers’ opinions on PrEP eligibility and safety		3		“It’s not the same if the person who’s telling you (the information) is someone who heard it from someone else, than a doctor who learned this from another doctor.” [IDI participant, Spanish, aged 36 years]	

^a^The n values in this column reflect the number of participants whose response was captured by the associated subcode within each theme. The total n of this column may exceed the total N for each theme because some participants offered responses to multiple subcodes.

^b^SMM: sexual minority men.

^c^LGBTQIA+: lesbian, gay, bisexual, transgender, queer, intersex, and asexual.

### Evaluation of Proposed App Functionalities

After discussing PrEP uptake barriers and facilitators with the interviewees, an IDI facilitator verbally described the SaludFindr goals of increased PrEP uptake, HIV testing, and condom use; used screenshots to present the main HealthMindr functions (periodic HIV risk assessments, customizable HIV testing, and PrEP care recommendations based on risk assessments, an HIV testing or PrEP service locator, and mail delivery of home HIV testing kits); and proposed 3 potential additional features specific for SaludFindr, as informed by discussions with our partner CBO, which included (1) a list of sexual health clinics known to provide patient-centered care to Latino SMM, (2) customized sex-positive motivational messages sent through in-app push notifications to incentivize PrEP uptake, and (3) an app function to facilitate discussions related to HIV and PrEP. Participants offered their general opinions on the acceptability of SaludFindr and then provided specific feedback for each of the above functions.

### Overall App Acceptability

The SaludFindr concept was well accepted and found to be very important by all participants. Most interviewees believed that the app could serve as an effective way to disseminate information related to HIV prevention and local PrEP care delivery among Latino groups. Without prompting, more than half (7/13, 54%) of the participants reported that the existence of the app would represent a sign that someone cares about their health, a way to feel seen, cared for, and included in society. This emergent theme was more common among those who were undocumented. Others noted that a tailored app would also help facilitate PrEP access by overcoming many of the intersectional stigmas they face in being Latino, gay, undocumented, or not speaking English:

There’s a lot of Latinos here (the Atlanta area). Most of us are undocumented. So, we don’t know where to go. We don’t know if we go to a clinic, what information they are going to ask for, how exposed we will be. So, if there’s a platform where we can be cared for or to get real information, so that’s going to be our...right hand, our cave, our place, right? I am Latino, I am gay, so if there’s an application where I can feel safe that I am in my community, I am with other Latino people looking for the same thing, looking to taking care of oneself, prevent any transmission. So, it would be very important, because nowadays, there’s nothing, I think.

IDI participant, Spanish, aged 27 years

### App Language and Tone

All participants who primarily spoke Spanish (8/13, 62%) noted that it would be important for all app content to be in Spanish, and even bilingual participants (2/13, 15%) considered that the availability of Spanish language for all app functions would be a way of signaling that the app is intentionally developed for Latino individuals. All participants agreed that the app should use lay terms, be clear and easy to understand, and avoid using medical terminology. The optimal overall app tone should be at a midpoint between formal (so it can be taken seriously) and informal (to make people feel comfortable):

Look, the Latino community here in the US is people who come to work, do you understand? So when they’re in a formal environment, they don’t feel comfortable. Of course, it shouldn’t be informal either, or very formal, in between those two.

IDI participant, Spanish, aged 27 years

### Periodic Health Assessments

Health assessments, administered at baseline and every quarter, would evaluate users’ HIV risk based on their current sexual behaviors. On the basis of their latest responses, SaludFindr would offer customized recommendations related to PrEP eligibility, HIV testing, and condom use. The assessments would also determine where participants are in their PrEP journey according to the Motivational PrEP Cascade Questionnaire [30] (PrEP precontemplation, PrEP contemplation, PrEParation, or PrEP action and initiation). Finally, the assessment would evaluate users’ PrEP-related stigma using a previously validated scale [31]. Nearly all participants (12/13, 92%) reported that they would use the periodic health assessments. They stated that these assessments would help them make decisions related to PrEP and HIV testing. Two participants recommended that assessments should be short and use yes or no or multiple-choice questions. They added that Latino individuals are tired of answering, and so tend to skip, long and repetitive health-related surveys:

If the quiz helps you get on that answer for people that are concerned at home, that are not encouraged...they think that they can’t go anywhere because they don’t have insurance, I think that would be great because I see the quiz as like, okay, I’m talking to a doctor, kind of.”

IDI participant, Spanish, aged 36 years

### Motivational Messages and Reminders

SaludFindr’s motivational messages and reminders were, overall, well accepted and considered helpful. Two participants specified that they would prefer if home screen messages and reminders would pop up as a generic message only, which then they could choose to pursue, or not, by opening the app. This was to mitigate concerns that overly specific pop-up notifications could tip off nearby people that they are using a sexual health app. One participant reported that receiving HIV prevention messages (instead of only reminders) would make him feel as if someone was looking out for his well-being, which would motivate him to keep using the app:

In a way, it’s beneficial, because sometimes one’s busy in many things and you forget because you have the app on silent. So, it’s good to receive reminders, information. It could also be information about events, I don’t know, for what the app was created for.

IDI participant, Spanish, aged 37 years

### Customized Sexual Health Clinics List

Our partner CBO reported that access to HIV prevention services is limited for Latino groups. As such, we decided to create a definition of a “Latino-and gay-friendly” clinic using IDI participants’ direct input. The participants identified 5 clinic characteristics as the defining criteria. These included (1) availability of Spanish language; (2) availability of free or low-cost PrEP care for uninsured and undocumented patients; (3) Latino representation in clinic staff and providers; (4) the clinic is perceived as a “safe space” for undocumented patients; and (5) LGBTQIA+ representation in the clinic through symbols (pride flag and clinic images), gay and transculturally concordant care and language, and availability of staff members who are part of LGBTQIA+ groups. Of these, availability of the Spanish language and free or low-cost PrEP were considered the most crucial, as all participants emphasized how important they were in facilitating access for Latino groups:

Those are the first things I think about when I think of accessible services for our communities: that they have community representation, that it’s an open environment for all, where you’re not judged, and accessible financially as well.

IDI participant, Spanish, aged 30 years

To develop SaludFindr’s tailored clinic list, we first compiled a list of all the clinics listed on PrEP locator [32-34] for the Atlanta Metropolitan Area. We then created a clinic criteria checklist based on the participant interviews. The staff reviewed the clinics’ websites and called the clinics to determine which of them fulfilled the prespecified criteria.

From the 47 clinics listed as PrEP providers on PrEP locator [32-34], we identified 17% (8/47) of clinics that fulfilled at least the 2 most crucial criteria—availability of the Spanish language and free or low-cost PrEP—and only 13% (6/47) of clinics that fulfilled all 5 criteria. The 8 clinics meeting the 2 most crucial criteria included 2 (25%) that were health department–based clinics, 5 (62%) that were community-based clinics funded by the Health Resources and Service Administration, and 1 (12%) that is part of a large safety net health care network in Atlanta. Most critically, only 25% (2/8) of the clinics were located in Gwinnett County, home to the highest percentage (22.2%) of Latinos in the state of Georgia [35]. These findings highlight the accessibility barrier to biomedical HIV prevention in Latino SMM and disclose the urgent need to increase the number of culturally concordant PrEP-providing clinics that serve this population.

### Products Delivery

We asked participants for their opinions on the acceptability and usability of HIV prevention items (home HIV testing kits, condoms, and personal lubricants) among Latino SMM. Approximately half (6/13. 46%) of the participants reported that they would use the home HIV testing kits as they considered them convenient. A common concern (7/13, 54%) was anxiety about making mistakes while conducting the test and not obtaining accurate results. They also highlighted the importance of including clear posttesting guidance in cases of positive results:

I get nervous even when I go get tested with a doctor, doing it myself, and then me having to read the result, I don’t know, it would give me a lot of stress and anxiety because, me, individually, I feel like I don’t know, in my mind, I’m going to do it wrong. Even if I get tested with a doctor, it’s—it makes me nervous, but doing it myself and then—me having to read the results, I don’t know, it would stress me out and give me anxiety because—only me, individually—I feel like, I don’t know, I’m going to do it wrong or something.

IDI participant, Spanish, aged 22 years

Approximately two-thirds (8/13, 62%) of the participants felt that having condoms and personal lubricants available through the app would be useful and could increase their use among people who already use condoms. Some noted that although condoms and lubricants are readily available for free at nightclubs and for sale at pharmacies, many Latino SMM would not take them because of fear of stigmatization. Home delivery of condoms was considered an asset as it would allow users to bypass this barrier.

### “Contact Us” Button

We explored different options to optimize discussions related to HIV prevention and PrEP within the app. All participants opted for private, anonymous, one-on-one conversations via written messages where they could ask questions. All participants agreed that the person interacting with the users should be a health care professional (physician, nurse, or health educator), as the opinion of peers was uniformly not trusted to be legitimate:

I think sometimes people get the wrong information. I mean they [peers] could suggest things, but if you want to get a real answer, I think it needs to be somebody that’s educated in it. Because that’s the whole point is that you’re getting information, the right information.

IDI participant, English, aged 44 years

### Potential Concerns

Most concerns expressed by participants were related to privacy, including anonymity while using the app and a need for clear communication about how app developers would ensure that the collected information would remain private. As noted earlier, participant suggestions related to privacy included wording home screen pop-up notifications to prevent others from understanding their importance and making the app symbol and name generic instead of relating it to sexual health, HIV, or PrEP.

## Discussion

### Principal Findings

In this study, the Latino SMM reported substantial social and structural barriers to PrEP uptake. Some of these barriers, including low perceived PrEP eligibility [36-39], intersectional stigma [40-42] (HIV, gay, and PrEP related), lack of insurance [43] or perceived cost [44], and PrEP misconceptions [45], are shared with other non-Latino groups (albeit with added complexities). Other barriers were more specific to the lives of Latino SMM. For example, participants emphasized the limited availability of trustworthy PrEP information and the difficulties accessing local sexual health services that, in most instances, were not intentionally designed to provide culturally concordant care to Latino SMM. Many of these barriers are potentially addressable through a tailored mHealth intervention with resources dedicated to responding to identified challenges.

Limited availability of reliable sources of PrEP information in Spanish and immigration-related factors result in great difficulties in accessing PrEP. This highlights the need to design and implement innovative strategies to disseminate information related to PrEP eligibility, PrEP safety, and how to access low-cost PrEP care locally, regardless of health insurance or immigration status. It is also essential to create safe spaces where Latino SMM can openly discuss PrEP access and other sexual health topics without fear of being stigmatized and safe processes for being able to receive information about PrEP without fear of invoking stigma.

As noted earlier, participants in our study described how peers are usually not a trusted source of PrEP information. Rhodes et al [46] launched a peer-based navigation intervention (HOLA) to increase HIV testing and condom use among Spanish-speaking Latino SMM and transgender women, which successfully increased HIV testing rates compared with a control group [46]. Careful selection and training of the navigators, in addition to a 12-month period of progressive trust building between the navigators and the participants, likely contributed to this success.

In our study, health care providers were perceived as trusted sources of information. Expanding the pool of PrEP-experienced, bilingual Latino health care providers in the Atlanta Metropolitan Area may help overcome this barrier and increase PrEP uptake. To achieve this, PrEP training for health care providers that currently provide education and care to Latino groups may be required. Important PrEP uptake facilitators were also identified; most were centered around increasing the representation of bilingual Latino health care providers and staff. Our findings align with previously published data, which emphasize the need to tailor existing PrEP delivery systems to meet community needs [41,47-50].

To address the identified barriers to and facilitators of PrEP uptake, we planned to develop an app that would increase self-awareness of PrEP eligibility; facilitate access to PrEP-related information, culturally responsive PrEP delivery care clinics, and direct HIV prevention tools; allow for private conversations about PrEP with a health care provider; and motivate users to uptake PrEP through sex-positive motivational messages. The use of a combination of prevention messages and access to HIV prevention services through a mobile app (M-cubed) has been shown to increase HIV testing and PrEP use within 3 months of app use in SMM in Atlanta, Detroit, and New York [51]. Such approaches demonstrate that tailored messaging has the potential for health impacts in the sphere of HIV prevention.

The app was very well accepted, with a special interest in those features that facilitate PrEP access, including the customized clinic list. On the basis of our search for PrEP-providing clinics that may facilitate access for Latino SMM in Metropolitan Atlanta, we concluded that only 4% (2/47) of the PrEP clinics in the Atlanta Metropolitan Area were accessible to Latino SMM and were in an area with the highest Latino population density of the state (Gwinnett County). Our findings affirm the urgent need to address the geographical barriers to PrEP uptake [52] and to develop strategies to make PrEP care clinics accessible to Latino communities.

### Limitations

Although we attempted to capture the opinions of diverse individuals, all participants were living in the Atlanta area, which is a newer immigrant destination than California, New York, Texas, and some other areas of the United States [53]. Consequently, the PrEP uptake needs and mobile app preferences identified in this study may not be generalizable to parts of the country where social support and PrEP access for Latino communities are more widely available [54]. In addition, most participants were foreign-born, limiting the applicability of these results to US-born Latino SMM. Finally, this study captured the opinions of Latino SMM only, which may differ in unknown ways from other Latinos at risk for HIV, including Latino transgender and gender nonbinary individuals who engage in condomless receptive anal sex.

### Comparison to Prior Work

Although the “My Choices” app, also adapted from HealthMindr, is designed to increase HIV testing and PrEP uptake among young SMM in Boston and New York city [55,56], the PrEPmate interactive SMS text messaging intervention is designed to increase PrEP retention and adherence among Latino youth [26]. SaludFindr would be, to our knowledge, the first mHealth intervention designed to address PrEP uptake specifically in *Latino SMM*. Furthermore, while the My Choices app was found to be acceptable and usable by participants during a 2-month technical pilot, approximately half (5/11, 45%) of the participants reported that the app assisted them in getting started in PrEP [55]. Only one-third (4/11, 36%) of the My Choices app participants were Latino and none of them lived in the southern United States, thus limiting the applicability of their findings in this group. The P3 (Prepared, Protected, emPowered) app study is ongoing and focuses on PrEP adherence and PrEP care persistence among young SMM and young transgender women who have sex with men [22]. These tailored approaches, while promising and important in addressing the needs of their target populations, have not been specifically designed with the needs of Latino SMM in mind, therefore suggesting that a tailored approach that focuses on PrEP *uptake* among Latino SMM is a critically unmet need, particularly because only 14% of PrEP-eligible Latino individuals were prescribed PrEP in the United States in 2019, compared with 66% among White individuals [57].

The next steps of the app will include tailoring each app feature based on the feedback received during the IDIs, creating an app development plan, and building a new app (ADAPT-ITT phase: production). We will then pilot test the app among Latino SMM to determine its acceptability and usability (ADAPT-ITT phase: testing). If found acceptable and usable, SaludFindr’s efficacy in increasing PrEP uptake among Latino SMM will be evaluated in the context of a randomized clinical trial.

### Conclusions

This study provides further evidence of the significant barriers to PrEP uptake that Latino SMM face; exposes the urgent need to increase the number of accessible PrEP-providing clinics for Latino SMM; and proposes an innovative, community-driven, and mobile technology–based tool as a future intervention to overcome some of these barriers.

## Abbreviations

ADAPT-ITT: Assessment, Decision, Adaptation, Production, Topical Experts–Integration, Training, Testing
CBO: community-based organization
COREQ: Consolidated Criteria for Reporting Qualitative Research
IDI: in-depth interview
LGBTQIA+: lesbian, gay, bisexual, transgender, queer, intersex, and asexual
mHealth: mobile health
PrEP: pre-exposure prophylaxis
SMM: sexual minority men
STI: sexually transmitted infection

## Data Availability

The data sets generated during or analyzed during this study are available from the corresponding author upon reasonable request.
